# The effect of diabetes mellitus on the association between measures of glycaemic control and ICU mortality: a retrospective cohort study

**DOI:** 10.1186/cc12572

**Published:** 2013-03-19

**Authors:** Marjolein K Sechterberger, Robert J Bosman, Heleen M Oudemans-van Straaten, Sarah E Siegelaar, Jeroen Hermanides, Joost BL Hoekstra, J Hans De Vries

**Affiliations:** 1 Department of Internal Medicine, Academic Medical Center, Meibergdreef 9, 1105AZ Amsterdam, the Netherlands; 2 Department of Intensive Care Medicine, Onze Lieve Vrouwe Gasthuis, Oosterpark 9, 1091AC Amsterdam, the Netherlands; 3 Department of Anesthesiology, Academic Medical Center, Meibergdreef 9, 1105AZ Amsterdam, the Netherlands

## Abstract

**Introduction:**

In critical illness, four measures of glycaemic control are associated with ICU mortality: mean glucose concentration, glucose variability, the incidence of hypoglycaemia (≤ 2.2 mmol/l) or low glucose (2.3 to 4.7 mmol/l). Underlying diabetes mellitus (DM) might affect these associations. Our objective was to study whether the association between these measures of glycaemic control and ICU mortality differs between patients without and with DM and to explore the cutoff value for detrimental low glucose in both cohorts.

**Methods:**

This retrospective database cohort study included patients admitted between January 2004 and June 2011 to a 24-bed medical/surgical ICU in a teaching hospital. We analysed glucose and outcome data from 10,320 patients: 8,682 without DM and 1,638 with DM. The cohorts were subdivided into quintiles of mean glucose and quartiles of glucose variability. Multivariable regression models were used to examine the independent association between the four measures of glycaemic control and ICU mortality, and for defining the cutoff value for detrimental low glucose.

**Results:**

Regarding mean glucose, a U-shaped relation was observed in the non-DM cohort with an increased ICU mortality in the lowest and highest glucose quintiles (odds ratio = 1.4 and 1.8, *P *< 0.001). No clear pattern was found in the DM cohort. Glucose variability was related to ICU mortality only in the non-DM cohort, with highest ICU mortality in the upper variability quartile (odds ratio = 1.7, *P *< 0.001). Hypoglycaemia was associated with ICU mortality in both cohorts (odds ratio non-DM = 2.5, *P *< 0.001; odds ratio DM = 4.2, *P *= 0.001), while low-glucose concentrations up to 4.9 mmol/l were associated with an increased risk of ICU mortality in the non-DM cohort and up to 3.5 mmol/l in the DM cohort.

**Conclusion:**

Mean glucose and high glucose variability are related to ICU mortality in the non-DM cohort but not in the DM cohort. Hypoglycaemia (≤ 2.2 mmol/l) was associated with ICU mortality in both. The cutoff value for detrimental low glucose is higher in the non-DM cohort (4.9 mmol/l) than in the DM cohort (3.5 mmol/l). While hypoglycaemia (≤ 2.2 mmol/l) should be avoided in both groups, DM patients seem to tolerate a wider glucose range than non-DM patients.

## Introduction

Hyperglycaemia, hypoglycaemia and increased glucose variability in critically ill patients are independently associated with ICU mortality [1-6]. In the last decade many clinical triallists have attempted to improve mortality rates through intensive insulin therapy. Unfortunately, these trials have produced conflicting data, with some of the studies showing lower and others higher mortality with strict glucose control, the latter possibly due to an increased incidence of hypoglycaemia [7-12]. There is consensus about the importance to avoid hypoglycaemia and many ICUs have therefore increased their lower glucose limit [13]. However, there is no consensus about the optimal target glucose range. In a previous database cohort study, we found an optimal mean glucose range of 6.7 to 8.4 mmol/l in a medical cohort and 7.0 to 9.4 mmol/l in a surgical cohort [14]. We additionally found that glucose concentrations that were low but above hypoglycaemic levels (between 2.3 and 4.7 mmol/l) were associated with increased ICU mortality [3]. Thus, in addition to the mean glucose concentration, glucose variability and hypoglycaemia, a fourth measure of glycaemic control - low glucose (2.3 to 4.7 mmol/l) - is associated with ICU mortality in the critically ill.

Underlying diabetes mellitus (DM) might affect the abovementioned associations. In a recent review we examined the current literature on glycaemic control and mortality in diabetic ICU patients and we found that, despite patients with DM having an increased risk of developing complications when admitted to the ICU, their risk of mortality is not increased [15]. In addition, ICU patients with DM have lower mortality in the higher mean glucose range compared with those without DM, although varying cutoff values were used [16-19]. Some studies found the opposite, with higher mortality rates for DM patients in the low-normal mean glucose range. However, these findings were unadjusted results only [18,20] and this relation was not significant after adjustment for severity of disease [16]. Furthermore, high glucose variability in ICU patients with DM seems to be less harmful than in patients without DM [21,22] although data are limited. Lastly, hypoglycaemia is associated with mortality in patients with and without DM [3,4,23], while the risk of hypoglycaemia is higher in patients with DM [4,24]. Altogether, some of the abovementioned findings are inconsistent and none of the reviewed studies evaluated all four measures of glycaemic control concomitantly.

The objective of this study was to determine whether the association between measures of glycaemic control - mean glucose, glucose variability (measured as the mean absolute glucose (MAG) change), the occurrence of hypoglycaemia (≤ 2.2 mmol/l) or low glucose (2.3 to 4.7 mmol/l) - and ICU mortality differs between patients without and with underlying DM in a large cohort of critically ill patients admitted to a general ICU of a teaching hospital in the Netherlands. We also explored the cutoff value for detrimental low glucose in both populations.

## Materials and methods

### Design and setting

The current study was conducted as a single-centre retrospective database cohort study in a 24-bed mixed surgical/medical ICU in a teaching hospital (Onze Lieve Vrouwe Gasthuis, Amsterdam, the Netherlands). All data were collected prospectively. All beds were equipped with a clinical information system (MetaVision; *i*MD*soft*, Tel Aviv, Israel) from which clinical and laboratory data were extracted. The nurse-to-patient ratio was on average 1:2, depending on the severity of disease. According to national guidelines this research is exempt from ethical approval because it is a retrospective study. The requirement for informed consent was waived because all data were anonymous and collected retrospectively.

### Glucose regulation protocol

A glucose regulation protocol, with a target blood glucose concentration of 4.0 to 7.0 mmol/l, was implemented in 2001 after the publication of the study by van den Berghe and colleagues [7]. The glucose regulation sliding scale algorithm was connected to the clinical information system and fully computerised with an integrated decision support module controlling the algorithm [25]. The glucose regulation protocol has been reported previously [2,3,14]. In April 2009, following the publication of the Normoglycaemia in Intensive Care Evaluation - Survival Using Glucose Algorithm Regulation investigators in 2009 [11], a new target blood glucose concentration of 5.0 to 9.0 mmol/l was instituted. To date, this new target blood glucose range is maintained in routine intensive care management.

### Cohort and data collection

Relevant data were extracted from the clinical information system concerning patients admitted to the ICU between January 2004 and June 2011. Readmissions, patients with a withholding care policy, and patients with < 3 glucose values during ICU admission were excluded. The assignment of each patient's diabetic status on ICU admission was based on the use of oral glucose-lowering drugs and/or insulin therapy. Demographic variables, admission diagnosis, glucose values, the occurrence of hypoglycaemia and ICU and hospital mortality rates were assessed. Severity of disease was assessed using the Acute Physiology and Chronic Health Evaluation (APACHE) II score on admission [26]. For each subsequent day of ICU admission, the Sequential Organ Failure Assessment score was assessed as a measurement of severity of disease [27]. The maximal Sequential Organ Failure Assessment score was determined for the patient's entire stay in the ICU [28].

### Glucose measurements

Glucose was measured from blood samples obtained from an arterial catheter using the Accu-chek (Roche/Hitachi, Basel, Switzerland). Results were automatically stored in the clinical information system. For each patient, mean glucose during admission was calculated from all glucose values measured during ICU admission. As markers for glucose variability, the MAG change [2] and the standard deviation were calculated per patient. Hypoglycaemia was defined as one or more glucose values ≤ 2.2 mmol/l, which is in accordance with previous trials [7,11]. Although our blood glucose target range in the initial years was between 4.0 and 7.0 mmol/l, we later found an association between the presence of a glucose value ≤ 4.7 mmol/l and ICU mortality [3]. Low glucose was therefore defined as the presence of at least one glucose value between 2.3 and 4.7 mmol/l.

### Statistical analyses

Continuous data are presented as mean (standard deviation) or median (interquartile range), as appropriate, and compared using Student's *t *test or the Mann-Whitney rank-sum test, respectively. Categorical data are presented as percentages and compared using the chi-square test. In accordance with our previous studies, mean glucose and glucose variability (MAG change) were categorised into equally sized quintiles [14] and quartiles [2] and were plotted against ICU mortality for the DM and non-DM cohorts separately.

The independent association between mean glucose and ICU mortality was examined using multivariable logistic regression analysis calculating odds ratios (ORs) with 95% confidence intervals (CIs). The quintile with the lowest mortality incidence was used as a reference. Based on clinical relevance and prognostic scoring, we adjusted for demographics (age, sex), severity of disease (using the APACHE II score), hypoglycaemia (≤ 2.2 mmol/l) and cardiothoracic surgery as the admission category. Cardiothoracic surgery was included as a covariate for several reasons: a generally lower mortality in this group compared with other surgical patients, a relatively low APACHE II score, a relatively short length of ICU stay and several different demographic and physiological characteristics of this group from the general ICU population, which could be reflected in differences in mean glucose concentration and glucose variability [29]. In an alternative model, adjustment was made for the occurrence of glucose values ≤ 4.7 mmol/l, which is also independently associated with mortality [3,30].

A second multivariable regression model was used to assess the independent association between glucose variability (MAG change) and ICU mortality, the quartile with lowest mortality incidence used as a reference. In this model the same potential confounders were used including the variable mean glucose. Furthermore, to assess the association between hypoglycaemia (≤ 2.2 mmol/l) and low glucose (2.3 to 4.7 mmol/l) and ICU mortality, unadjusted and adjusted ORs with 95% CIs were calculated, the latter using a third multivariable regression model adjusted for age, sex, severity of disease, cardiothoracic surgery and sepsis as admission diagnoses.

In both cohorts, we also assessed the cutoff value for detrimental low glucose, by performing the latter analysis for different blood glucose cutoff values. Additionally, when we adjusted the logistic regression models for the change in target glucose range in the studied period, no change in our results was observed (data not shown). All statistical analyses were performed in SPSS 18.0 (SPSS Inc, Chicago, IL, USA).

## Results

From 11,901 ICU admissions, 10,320 patients were selected for analyses after excluding 842 readmissions, 105 patients with a withholding care policy, and 714 patients with < 3 glucose measurements. The remaining cohort consisted of 8,682 (84.2%) patients who did not have DM at the time of ICU admission (non-DM cohort) and 1,638 (15.8%) patients who had DM at the time of ICU admission (DM cohort). The percentage of medical and surgical ICU admissions in the entire cohort was 26% and 74%. The non-DM:DM ratio within these groups was 9:1 in patients with a medical ICU admission diagnosis and 4:1 in patients with a surgical ICU admission diagnosis. Table 1 illustrates patient characteristics of the entire study population as well as the differences between the non-DM cohort and the DM cohort.

**Table 1:** Characteristics, glucose and treatment variables for patients without/with diabetes mellitus and the total cohort

	No diabetes (*n *= 8,682)	Diabetes (*n *= 1,638)	*P *valueª	Total cohort (*n *= 10,320)
				
Age (years)	65 ± 13	68 ± 10	< 0.001	65 ± 13
Male sex	5804 (67)	1,032 (63)	0.003	6,836 (66)
Body mass index (kg/m^2^)	27 ± 14	29 ± 5	< 0.001	27 ± 13
APACHE II score on admission	16 (13 to 21)	16 (13 to 20)	0.006	16 (13 to 21)
Maximum SOFA score during admission^b^	6 (5 to 8)	6 (5 to 7)	0.09	6 (5 to 8)
ICU stay (hours)	26 (20 to 66)	23 (19 to 49)	< 0.001	25 (20 to 64)
Died in the ICU	622 (7)	73 (5)	< 0.001	695 (7)
Died in the hospital	994 (11)	144 (9)	0.001	1,138 (11)
Medical admissions	2,444 (28)	266 (16)	< 0.001	2,710 (26)
Surgical admissions	6,238 (72)	1,372 (84)	< 0.001	7,610 (74)
Cardiothoracic surgery patients	4,877 (56)	1,214 (74)	< 0.001	6,091 (59)
APACHE II admission category				
Cardiovascular	5,776 (67)	1,338 (82)	< 0.001	7114 (69)
Sepsis	628 (7)	93 (6)	0.02	721 (7)
After cardiac arrest	534 (6)	37 (2)	< 0.001	571 (6)
Gastrointestinal	474 (5)	43 (3)	< 0.001	517 (5)
Haematological	18 (0)	1 (0)	0.205	19 (0)
Renal	60 (1)	9 (1)	0.519	69 (1)
Metabolic	81(1)	14 (1)	0.761	95 (1)
Neurological	266 (3)	12 (1)	< 0.001	278 (3)
Respiratory	845 (10)	91 (6)	< 0.001	936 (9)
Glucose values per patient	12 (7 to 27)	14 (11 to 28)	< 0.001	13 (8 to 28)
Overall glucose (mmol/l)	8.0 ± 1.7	8.0 ± 1.6	0.577	8.0 ± 1.6
Morning glucose (mmol/l)	7.6 ± 2.0	7.0 ± 2.0	< 0.001	7.5 ± 2.0
Mean absolute glucose change (mmol/l/hour)	0.6 (0.4 to 0.8)	0.8 (0.6 to 1.0)	< 0.001	0.7 (0.4 to 0.9)
Standard deviation (mmol/l)	1.7 (1.3 to 2.3)	2.1 (1.6 to 2.7)	< 0.001	1.8 (1.4 to 2.4)
Incidence hypoglycaemia ≤ 2.2 mmol/l^c^	310 (4)	57 (4)	0.856	367 (4)
Incidence glucose value 2.3 to 4.7 mmol/l^c^	3,715 (43)	901 (55)	< 0.001	4,616 (45)
Use of insulin	6,686 (77)	1,610 (98)	< 0.001	8,296 (80)
Insulin dose (IU/hour)	2.2 (1.7 to 3.1)	2.8 (2.0 to 4.0)	< 0.001	2.3 (1.8 to 3.3)
Use of vasopressor drugs	8,020 (92)	1,551 (95)	0.001	9,571 (93)
Use of corticosteroids	8,561 (99)	1,636 (100)	< 0.001	10,197 (99)
Mechanical ventilation^d^	8,039 (93)	1,539 (94)	0.050	9,578 (93)
Continuous veno-venous haemofiltration	690 (8)	116 (7)	0.231	806 (8)

Data presented as mean ± standard deviation, *n *(%) or median (interquartile range). APACHE, Acute Physiology and Chronic Health Evaluation; SOFA, Sequential Organ Failure Assessment. ^a^Based on Student's *t *test or the Mann-Whitney rank-sum test (continuous data), or the chi-square test (categorical data), comparing patients with and without diabetes. ^b^Maximum score during admission, calculated from the total individual scores calculated each ICU day. ^c^Patients who experienced at least one hypoglycaemia or glucose value between 2.3 and 4.7 mmol/l. ^d^In the first 24 hours of ICU admission.

### Association between mean glucose concentration and ICU mortality

**Figure 1:**
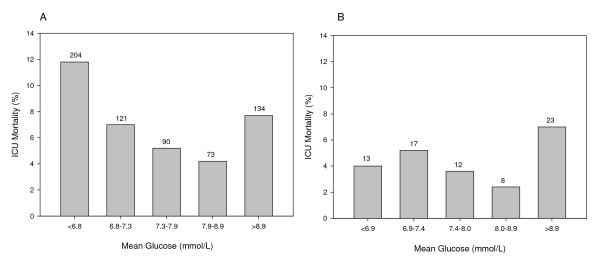
**ICU mortality per quintile of mean glucose in the nondiabetes mellitus and diabetes mellitus cohorts**. ICU mortality (%) per quintile of mean glucose in **(A) **the nondiabetes mellitus cohort and **(B) **the diabetes mellitus cohort. Numbers above bars indicate the number of deaths per mean glucose quintile.

**Figure 2:**
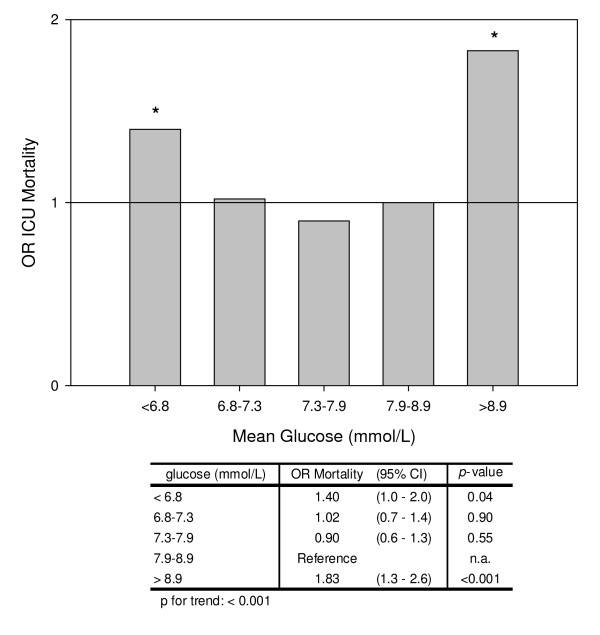
**Odds ratio for ICU mortality per quintile of mean glucose in the nondiabetes mellitus cohort**. All odds ratios (ORs) were calculated per quintile of mean glucose and adjusted for age, sex, Acute Physiology and Chronic Health Evaluation II admission score, cardiothoracic surgery as admission diagnosis and the occurrence of hypoglycaemia (≤ 2.2 mmol/l). *P < 0.05. CI, confidence interval.

Figure 1 demonstrates the quintiles of mean glucose ranges per cohort (non-DM cohort: < 6.8, 6.8 to 7.3, 7.3 to 7.9, 7.9 to 8.9, > 8.9 mmol/l; DM cohort: < 6.9, 6.9 to 7.4, 7.4 to 8.0, 8.0 to 8.9, > 8.9 mmol/l) and corresponding ICU mortality rates. This resulted in a U-shaped relationship between mean glucose and ICU mortality in the non-DM cohort, with high ICU mortality in the lowest and highest glucose quintile (11.8% and 7.7%). Multivariable logistic regression analysis in the non-DM cohort showed that mean glucose values in the lowest and highest quintiles were associated with a significantly higher OR for ICU mortality compared with the quintile with the lowest ICU mortality (Figure 2). This was supported by a significant nonlinear relationship between mean glucose and ICU mortality (*P *for trend < 0.001). When we adjusted the logistic regression model for the occurrence of glucose values ≤ 4.7 mmol/l, the OR for ICU mortality in the lowest quintile no longer reached significance in the non-DM cohort (OR = 1.3, 95% CI = 0.9 to 1.8, *P *= 0.17). The increased ICU mortality in the non-DM cohort in the lower part of the U-curve therefore seems to be due to the relation between glucose values ≤ 4.7 mmol/l and ICU mortality. In contrast, no clear pattern was found in the DM cohort in unadjusted (Figure 1B) or multivariate analysis (data not shown).

### Association between glucose variability and ICU mortality

**Figure 3:**
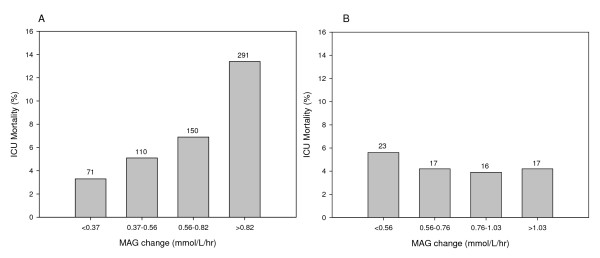
**ICU mortality per mean absolute glucose change quartile in non-diabetes mellitus and diabetes mellitus cohorts**. ICU mortality (%) per mean absolute glucose change (MAG) quartile in **(A) **the nondiabetes mellitus cohort and **(B) **the diabetes mellitus cohort. Numbers above bars indicate number of deaths per mean absolute glucose change quartile.

**Figure 4:**
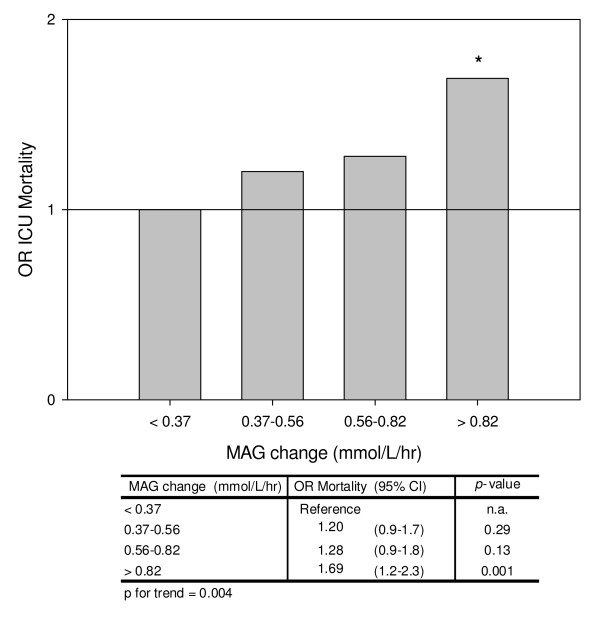
**Odds ratio for ICU mortality over mean absolute glucose quartiles in the nondiabetes mellitus cohort**. All odds ratios (ORs) were calculated per quartile of mean absolute glucose (MAG) change and adjusted for age, sex, Acute Physiology and Chronic Health Evaluation II admission score, mean glucose, cardiothoracic surgery as admission diagnosis and the occurrence of hypoglycaemia (≤ 2.2 mmol/l). *P < 0.05. CI, confidence interval.

The ranges of MAG change per quartile (non-DM cohort: < 0.37, 0.37 to 0.56, 0.56 to 0.82, > 0.82 mmol/l/hour; DM cohort: < 0.56, 0.56 to 0.76, 0.76 to 1.03, > 1.03 mmol/l/hour) and corresponding ICU mortality per cohort are shown in Figure 3. This resulted in a linear relationship in the non-DM cohort, with the highest mortality rate seen in the upper MAG quartile (13.4%). Multivariable logistic regression analysis for the non-DM cohort is displayed in Figure 4; the OR for ICU mortality was highest in the upper MAG change quartile (OR = 1.69, *P *= 0.001). This was supported by a significant relationship between MAG quartiles and ICU mortality (*P *for trend = 0.004). In contrast, in the DM cohort no clear pattern was found in unadjusted (Figure 3B) or multivariate analysis (data not shown).

### Association between hypoglycaemia and low glucose and ICU mortality

The percentage of patients who experienced at least one episode of hypoglycaemia (≤ 2.2 mmol/l) was similar in both cohorts (Table 1). Low glucose (2.3 to 4.7 mmol/l) occurred more frequently in the DM cohort. The increase in glucose target range as introduced in 2009 decreased the percentage of patients who experienced both hypoglycaemia (before 3.3%; after 0.3%) and low glucose (before 36.3%; after 8.4%).

ICU mortality rates for hypoglycaemia were 29.7% and 21.1% in the non-DM and DM cohorts, respectively. Unadjusted ORs of hypoglycaemia (≤ 2.2 mmol/l) for ICU mortality in the occurrence of hypoglycaemia were 6.2 (95% CI = 4.8 to 8.1, *P *< 0.001) in the non-DM cohort and 6.6 (95% CI = 3.3 to 13.1, *P *< 0.001) in the DM cohort. In logistic regression analysis, adjusted for potential confounders (see above), the OR of hypoglycaemia for ICU mortality was still significant in both cohorts (non-DM cohort: OR = 2.5, 95% CI = 1.8 to 3.4, *P *< 0.001; DM cohort: OR = 4.2, 95% CI = 1.8 to 10.1, *P *= 0.001).

ICU mortality rates for low glucose (2.3 to 4.7 mmol/l) were 13.1% and 5.2% in the non-DM and DM cohorts, respectively. The OR of low glucose for ICU mortality was significant in the non-DM cohort (unadjusted OR = 5.3, 95% CI = 4.4 to 6.4, *P *< 0.001; adjusted OR = 1.5, 95% CI = 1.2 to 1.9, *P *< 0.001). When exploring the cutoff value for detrimental low glucose in the non-DM cohort, we found that lowest blood glucose concentrations up to 4.9 mmol/l were associated with an increased risk for ICU mortality (adjusted OR = 1.3, 95% CI = 1.1 to 1.7, *P *= 0.01). In contrast, when exploring the cutoff value for detrimental low glucose in the DM cohort, we found that lowest blood glucose concentrations up to 3.5 mmol/l were associated with an increased risk of ICU mortality (adjusted OR = 2.1, 95% CI = 1.2 to 3.7, *P *= 0.01). With glucose values between 3.5 and 4.7 mmol/l, no significant effect on the OR for ICU mortality was found. Poisson regression analysis, which we used in a previous study to adjust for daily Sequential Organ Failure Assessment score over time [3], amounted to similar results (data not shown).

## Discussion

In this retrospective database cohort study evaluating the association of four measures of glycaemic control and ICU mortality concomitantly, we found striking differences between the non-DM cohort and the DM cohort. In the non-DM cohort, ICU mortality was significantly related to all four measures of glycaemic control: mean glucose, glucose variability, the occurrence of hypoglycaemia (≤ 2.2 mmol/l) and low glucose concentrations up to 4.9 mmol/l. In contrast, in the DM cohort, only the occurrence of hypoglycaemia (≤ 2.2 mmol/l) and low-glucose concentrations up to 3.5 mmol/l were significantly associated with ICU mortality, while mean glucose and glucose variability were not. The presence of DM thus seems to affect the association between glucose control and ICU mortality.

Our findings support the results of previous studies that have focused on understanding the association between the presence of DM at ICU admission, glycaemia, and ICU mortality [7,8,16-19,31,32]. In all these studies, a stronger association between hyperglycaemia and ICU mortality was found in patients without DM, in comparison with patients with DM.

Hypoglycaemia has been found to be a risk factor of mortality in patients without and with DM in the literature [3,4,7,8,30,33,34]. Of note, different cutoff values were used to define hypoglycaemia, ranging from ≤ 2.2 mmol/l [4,35] up to ≤ 4.7 mmol/l [3,33]. We also found a significant independent association between hypoglycaemia (≤ 2.2 mmol/l) and ICU mortality, in both the non-DM and DM cohorts. However, the association between low glucose (2.3 and 4.7 mmol/l) and ICU mortality was only significant in the non-DM cohort, not in the DM cohort. When exploring the cutoff value for detrimental low glucose in the present cohort, we found that lowest blood glucose concentrations up to 4.9 mmol/l were associated with an increased risk of ICU mortality in the non-DM cohort, and 3.5 mmol/l in the DM cohort. The cutoff value in the non-DM cohort is in line with our previous study, in which we found that lowest glucose values up to 4.7 mmol/l were associated with significant increased ICU mortality [3]. Furthermore, this cutoff value is supported by the finding that the higher mortality in the lower half of the U-shaped curve (< 6.8 mmol/l) in the non-DM cohort is mainly determined by the occurrence of glucose values ≤ 4.7 mmol/l and less by the glucose range between 4.7 and 6.8 mmol/l. The cutoff value for detrimental low glucose we found in our DM cohort (≤ 3.5 mmol/l) is also in line with the literature [23,30]. Both studies found that glucose concentrations ≤ 3.9 mmol/l were significantly associated with mortality in a subgroup of DM patients. Altogether, we can conclude that the cutoff value for detrimental low glucose is lower in the DM population than in the non-DM population.

The association between glucose variability and ICU mortality in patients without and with DM was studied previously [22]. In this observational study of 4,084 patients (including 942 DM patients), a strong association of glucose variability - expressed as the coefficient of variation (standard deviation/mean glucose level) - with mortality was found in patients without DM, while, in concordance with our study, no association was found in patients with DM [22]. Of note, this measure of glucose variability does not take order and time into account.

Several explanations can be considered for the different associations between glycaemic control and ICU mortality in patients without and with pre-existing DM. We previously suggested that adaptation to hyperglycaemia might be a key mechanism [15]. Acute hyperglycaemia and inflammation induce oxidative stress, which causes endothelial damage [36]. In patients without DM, cellular adaptation mechanisms will be activated for the first time in the acute care setting, whereas patients with DM could already have adapted to these insults during their years with DM and therefore better tolerate episodes of hyperglycaemia in an acute care setting. In addition, cellular adaptation to recurrent hypoglycaemia is also a well-established phenomenon [37-39]. Although speculative, adaptation to low glucose will already be present in patients with DM and might explain why patients with DM can withstand relatively low glucose values better.

Our results should be viewed in light of the study's strengths and limitations. Strengths of our study include the large number of ICU patients and that glucose values were captured automatically, which prevents transcription errors. Furthermore, this is the first study examining all four markers of glycaemic control in a non-DM cohort and a DM cohort simultaneously. Also, we used a time-based metric for glucose variability and we explored multiple cutoff values for hypoglycaemia. Potential limitations of the study are that it is a single-centre study and retrospective in design, and thus is potentially subject to systematic error and bias. However, all data were prospectively collected and independently measured. Moreover, the findings are robust and internally consistent.

As in all studies in this field, our definition for a patient's diabetic status may be nonrepresentative. Unfortunately, glycosylated haemoglobin testing was not performed before ICU admission and we were unable to make a distinction between type 1 and type 2 DM patients. In addition, we were not able to distinguish between diabetes patients with good and poor chronic control, who may become hyperglycaemic due to acute illness. Whether this might affect the optimal glucose target for the DM cohort remains unknown.

Another limitation was that we were not able to distinguish between spontaneous (illness-related) and treatment-induced hypoglycaemia or variability. However, other studies could make this distinction. Finfer and colleagues reported that patients who had encountered severe or moderate hypoglycaemia while not being treated with insulin were at an increased mortality risk [23]. But they also demonstrated that, although to a lesser extent, insulin-induced hypoglycaemia was associated with an increased risk for ICU death. In contrast, Kosiborod and colleagues only reported a high risk for mortality in patients hospitalised with acute myocardial infarction who developed hypoglycaemia spontaneously. Iatrogenic hypoglycaemia after insulin therapy was not associated with higher mortality risk [40].

Furthermore, in our cohort, most patients were admitted for cardiothoracic surgery; we corrected for this potential confounder in our regression analyses and still found significantly increased ICU mortality in the lowest and highest mean glucose quintiles and in the highest glucose variability quartile in the non-DM cohort. Moreover, the high amount of cardiothoracic surgery patients in the studied cohort may also have contributed to the high administration level of corticosteroids. In our hospital, as in many European hospitals (but not in most North American cardiac surgical centres), corticosteroid administration during cardiac surgery is part of routine care. All patients who were in shock or had sepsis or systemic inflammatory response syndrome also received corticosteroids. This could possibly limit the external validity of this single-centre study.

In our analyses of glucose variability, we did not correct for the frequency of glucose measurements during ICU admission. However, we did correct for severity of disease, which in itself is clearly correlated with the frequency of glucose measurements and ICU mortality. Furthermore, the concern that the frequency of blood glucose measurements may influence the relation between the MAG and ICU mortality has been previously discussed [41]. MAG is independent of the number of measurements, as long as blood glucose keeps changing at a constant rate. The MAG only increases when there is actually more glucose variability. The possibility to capture variability, if there is any, increases when the number of glucose measurements is increased. However, this can be said for all measures of glucose variability and this is not unique for the MAG change.

A limitation of our correction for severity of disease is the use of the APACHE II score. Although the validation of the use of APACHE II score to predict mortality in cardiac surgery patients is lacking, this adjustment is the best available method [29]. Finally, because of the observational nature of the study, no proof of causation can be derived from the abovementioned associations between glycaemic control and ICU mortality.

## Conclusion

This retrospective database cohort study shows that the presence of DM affects the association between three out of four measures of glycaemic control and ICU mortality. Mean glucose and high glucose variability were associated with ICU mortality in the non-DM cohort but not in the DM cohort, whereas hypoglycaemia (≤ 2.2 mmol/l) was associated with ICU mortality in both. We additionally found a higher cutoff value for detrimental low glucose in our non-DM cohort (4.9 mmol/l) than the DM cohort (3.5 mmol/l). Glucose concentrations ≤ 4.9 mmol/l should therefore be avoided in the non-DM cohort, while DM patients seem to tolerate a wider glucose range. Further studies should examine whether new technologies - that is, continuous glucose monitoring technology - could be of use for clinicians to improve glycaemic control.

## Key messages

• The presence of DM affects the association between three out of four measures of glycaemic control and ICU mortality.

• Mean glucose relates to ICU mortality by a U-shaped curve in the non-DM population, whereas no clear association was found in the DM population.

• High glucose variability is only related to ICU mortality in the non-DM cohort.

• The occurrence of hypoglycaemia (≤ 2.2 mmol/l) is related to ICU mortality in both populations and should undoubtedly be avoided.

• The cutoff value for detrimental low glucose in the non-DM population (4.9 mmol/l) seems to be higher than in the DM population (3.5 mmol/l).

## Abbreviations

APACHE: Acute Physiology and Chronic Health Evaluation; CI: confidence interval; DM: diabetes mellitus; MAG: mean absolute glucose; OR: odds ratio.

## Competing interests

The authors declare that they have no competing interests.

## Authors' contributions

MKS participated in the design of the study, performed the statistical analysis, and wrote the manuscript. HMO-vS, SES, JH and JBLH participated in the design of the study, contributed to the interpretation of the data, and revised the manuscript critically for important intellectual content. RJB participated in the design of the study, performed acquisition of the data, contributed to the interpretation of the data, and revised the manuscript for important intellectual content. JHDV participated in the design of the study, contributed to the interpretation of the data, and participated in the writing of the manuscript. All authors read and approved the final manuscript.
